# The Role of Heparin in Postural Orthostatic Tachycardia Syndrome and Other Post-Acute Sequelae of COVID-19

**DOI:** 10.3390/jcm13082405

**Published:** 2024-04-20

**Authors:** Elisabeth Gómez-Moyano, Javier Pavón-Morón, Jorge Rodríguez-Capitán, Daniel Bardán-Rebollar, Teresa Ramos-Carrera, Aurora Villalobos-Sánchez, Iván Pérez de Pedro, Francisco J. Ruiz-García, Javier Mora-Robles, Almudena López-Sampalo, Miguel A. Pérez-Velasco, Maria-Rosa Bernal-López, Ricardo Gómez-Huelgas, Manuel Jiménez-Navarro, Miguel Romero-Cuevas, Francesco Costa, Alicia Trenas, Luis M. Pérez-Belmonte

**Affiliations:** 1 Servicio de Dermatología, Hospital Regional Universitario de Málaga, 29010 Málaga, Spain; eligm80@hotmail.com; 2 Servicio de Cardiología, Hospital Universitario Virgen de la Victoria, 29010 Málaga, Spain; javier.pavon@ibima.eu (J.P.-M.); mjimeneznavarro@gmail.com (M.J.-N.); mromcue@gmail.com (M.R.-C.); 3 Centro de Investigación en Red de Enfermedades Cardiovasculares (CIBERCV), IBIMA-Plataforma BIONAND, Universidad de Málaga (UMA), 29010 Málaga, Spain; luismiguelpb@hotmail.com; 4 Servicio de Hematología, Hospital Regional Universitario de Málaga, 29010 Málaga, Spain; 5 Servicio de Neumología, Hospital de la Serrania de Ronda, 29400 Ronda, Spain; 6 Servicio de Medicina Interna, Hospital Regional Universitario de Málaga, 29010 Málaga, Spainivanpdp@hotmail.com (I.P.d.P.); almu_540@hotmail.com (A.L.-S.); rosa.bernal@ibima.eu (M.-R.B.-L.); ricardogomezhuelgas@hotmail.com (R.G.-H.); 7 Servicio de Medicina Nuclear, Hospital Regional Universitario de Málaga, 29010 Málaga, Spain; javierruiz59@hotmail.com; 8 Servicio de Cardiología, Hospital Regional Universitario de Málaga, 29010 Málaga, Spain; 9 Centro de Investigación en Red Fisiopatología de la Obesidad y la Nutrtición (CIBERObn), IBIMA-Plataforma BIONAND, Universidad de Málaga (UMA), 29010 Málaga, Spain; 10 Department of Biomedical and Dental Sciences and Morphological and Functional Imaging, University of Messina, A.O.U. Policlinic ‘G. Martino’, Via C. Valeria 1, 98165 Messina, Italy; dottfrancescocosta@gmail.com; 11 Servicio de Medicina Interna, Área Sanitaria Norte de Málaga, Hospital de Antequera, 29200 Antequera, Spain; aliciatrenas@hotmail.es; 12 Servicio de Medicina Interna, Hospital Helicópteros Sanitarios, 29660 Marbella, Spain

**Keywords:** COVID-19, heparin, post-acute sequelae, postural orthostatic tachycardia syndrome, microvascular thrombosis

## Abstract

The therapeutic management and short-term consequences of the coronavirus disease 2019 (COVID-19) are well known. However, COVID-19 post-acute sequelae are less known and represent a public health problem worldwide. Patients with COVID-19 who present post-acute sequelae may display immune dysregulation, a procoagulant state, and persistent microvascular endotheliopathy that could trigger microvascular thrombosis. These elements have also been implicated in the physiopathology of postural orthostatic tachycardia syndrome, a frequent sequela in post-COVID-19 patients. These mechanisms, directly associated with post-acute sequelae, might determine the thrombotic consequences of COVID-19 and the need for early anticoagulation therapy. In this context, heparin has several potential benefits, including immunomodulatory, anticoagulant, antiviral, pro-endothelial, and vascular effects, that could be helpful in the treatment of COVID-19 post-acute sequelae. In this article, we review the evidence surrounding the post-acute sequelae of COVID-19 and the potential benefits of the use of heparin, with a special focus on the treatment of postural orthostatic tachycardia syndrome.

## 1. Introduction

The therapeutic management and short-term consequences of the coronavirus disease 2019 (COVID-19) caused by the severe acute respiratory syndrome coronavirus 2 (SARS-CoV-2) are well known. However, the COVID-19 post-acute sequelae (PASC) are less studied and represent a public health problem worldwide [1].

An important proportion of patients affected by COVID-19 at the beginning of the pandemic could not benefit from the current treatment strategies due to the lack of robust evidence. Hence, a great number of patients now present COVID-19 PASC. A meta-analysis estimated the prevalence of post-COVID-19 condition at 43% (95% confidence interval 39–46%) [2]. In these patients, several mechanisms have been proposed as pathological mechanisms, such as immune dysregulation, a procoagulant state, and persistent microvascular endotheliopathy related to crafyptic SARS-CoV-2 tissue reservoirs that could participate in the presence of microvascular thrombosis in multiple tissues [3,4,5,6]. One frequent sequela presented in post-COVID-19 patients is postural orthostatic tachycardia syndrome. Hypercoagulability, autoimmunity, and endothelial dysfunction mechanisms have been implicated in its physiopathology [3,4]. These mechanisms can determine the thrombotic sequelae nature of COVID-19 and the need for early anticoagulation therapy [6].

The use of heparin has been associated with a reduction in all-cause mortality in hospitalized, non-critically ill patients with COVID-19 in a recent meta-analysis [7]. Heparin, beyond its use as an anticoagulant, has been described as a treatment with several potential beneficial properties such as immunomodulatory, antiviral, endothelial, and vascular resistance effects through different mechanisms. These properties could be helpful in the treatment of COVID-19 post-acute sequelae [8].

In this article, we review the evidence surrounding the post-acute sequelae of COVID-19, the presence of postural orthostatic tachycardia syndrome, the pathological mechanism implicated, and the potential benefits of the use of heparin.

## 2. Materials and Methods

**Figure 1:**
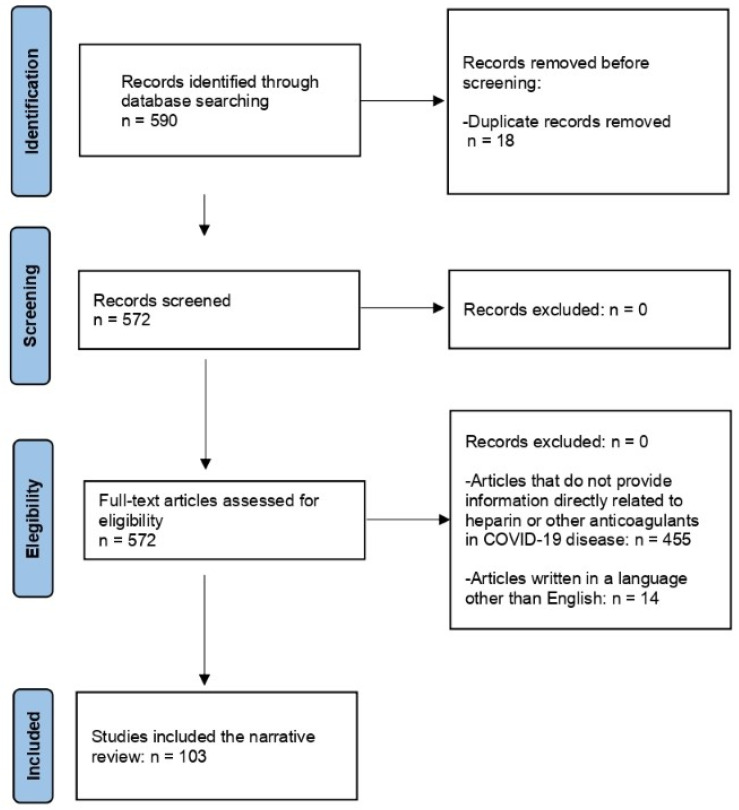
Flow chart of article inclusion.

A narrative literature review was performed based on the PubMed electronic bibliographic database, Web of science, Scopus, and EMBASE. The following descriptors were used in the search engines’ advanced tools: “COVID-19”, “SARS-CoV-2”, “heparin”, “low molecular weight heparin”, “post-acute sequelae of SARS-CoV-2 syndrome”, “PASC”, “post COVID syndrome”, “postural orthostatic tachycardia syndrome”, “POTS”, “dysautonomia”, “autonomic dysfunction”, “muscarinic receptor”, “microclot”, “endotheliitis”, “immunothrombosis”, and “heparanase”. The filters used were as follows: free full text, case reports, clinical study, multicenter study, observational study, humans, systematic review, and meta-analysis sorted by most recent. Finally, a manual selection of the bibliographic references was carried out. Articles with a central topic diverging from heparin or COVID-19 infection were excluded from the revision. Articles that were not in English were also excluded. The search was conducted in April 2023. Figure 1 shows flow chart of the process of article inclusion.

## 3. Post-Acute Sequelae of COVID

The synchronous presence of inflammation and thrombosis may be part of a response from the host’s defense against invading pathogens. This response is caused by a dysregulation among different molecules that participate in the immunothrombosis process, such as tissue factors, histones, and factors of the contact system, as well as implication cells, including platelets, endothelial cells, and neutrophil extracellular traps [9,10].

Inmmunothrombosis has been described as the clue mechanism implicated in the pathophysiology of COVID-19 at different clinical stages, including those in patients who develop long-lasting complications [11,12]. Numerous studies have implicated the presence of endothelial damage, microthrombosis, and inflammation in the pathogenesis of PASC associated with COVID-19 [13,14,15,16,17,18,19,20,21,22,23,24,25,26,27] through an in situ thrombotic microangiopathy and a complex immune inflammatory cascade, especially in the pulmonary vascular bed [21], which is described as microvascular COVID-19 lung vessels obstructive thromboinflammatory syndrome (MicroCLOTS) [23].

Several lung perfusion phenotypes have been described in COVID-19, including an atypical combination of classical segmental affection and distal small-vessel-related deficits, which might be subtle and easily overlooked [21].

PASC have been associated with multisystem tissue damage, including cardiopulmonary disorders, encephalitis, hepatobiliary damages, gastrointestinal dysregulation, neuromuscular syndromes, neuropsychiatric disorders, renal failure, stroke, and vascular endothelial dysregulation as cause of the molecular mimicry between pathogen and host proteins [28,29]. Due to this participation of multiple systems, its presentation can vary widely, from acute multiorgan SARS-CoV-2 injury to the persistence of the replicating virus reservoirs, the reactivation of latent pathogens such as Epstein–Barr and herpes viruses in the COVID-19 immune-dysregulated tissue environment, SARS-CoV-2 interactions with host microbiome/virome communities, clotting/coagulation dysregulation, the presence of microclots, dysfunctional brainstem/vagus nerve signaling, autonomic dysfunction, or autoimmunity disorders [29].

The pathogenesis of COVID-19 is complex, and three physiological systems have been directly involved: the kinin–kallikrein system, the renin–system angiotensin, and the coagulation system coexpressed with the angiotensin-converting enzyme 2 (ACE2) receptor in alveolar cells [30]. SARS-CoV-2 binds to the ACE2 receptor through its spike protein and penetrates different types of cells, such as epithelial cells and macrophages [31]. The overexpression of the ACE2 receptor may dysregulate these systems and cause cardiovascular instability (renin–angiotensin system), acute inflammatory pulmonary edema (kinin–kallikrein system), and thromboembolism (coagulation system) [30]. SARS-CoV-2 infects ACE2-expressing pneumocyte type II cells, which act as epithelial immune cells and produce tumor necrosis factor (TNF) and interleukins (IL-6, IL-1β, and Monocyte Chemoattractant Protein-1-MCP-1-). Moreover, these ACE-2 receptors are also expressed in the myocardium, and mice models have shown that the SARS-CoV-2 may regulate ACE2 activity and produce myocardial inflammation, as reported in the Castiello meta-analysis [32]. When SARS-CoV-2 binds to the ACE2 receptor and enters the cell, a decreased density of the receptor on the vascular tissue ensues, leading to a negative regulation of ACE2 activity and a secondary accumulation of angiotensin 2, causing vasoconstriction, pro-inflammatory effects, and tissue fibrosis [33].

The disproportionate activity of angiotensin 2 and the increased release of inflammatory cytokines IL-1 and IL-6 by the activated macrophages generate the endothelial activation and increase the permeability and the co-expression of adhesion molecules, favoring the prothrombotic phenotype [34].

The cytokine storm produces macrophage polarization from M2 to M1 macrophages, T-cell cytotoxicity defects, complement activation [35], and increased neutrophil extracellular traps (NETs) [36]. The accumulation of NETs leads to a greater activation of factor XII and increases the DNA and H4 histones [37]. Histones have been described to contribute to microvascular thrombosis and competitively inhibit plasmin to delay fibrinolysis. Sequelae of COVID-19 PASC are accompanied by increased levels of antiplasmin [22].

On the other hand, the viral replication is maintained with stress granules, and, additionally, the antigens are presented to naïve T-lymphocytes by the antigen-presenting cells, which show cardiotropism by the hepatocyte growth factor (HGF). HGF binds to c-Met, an HGF receptor on T-lymphocytes, and the myocardial damage is caused by cell-mediated cytotoxicity [38]. HGF upregulation has been associated with the moderate and severe stages of COVID-19 [39].

In an environment with a persistent presence of the virus, the endocrine system possesses the required ACE2 receptor and the presence of transmembrane serine protease 2 (TMPRSS2) (a protein regulated by androgens), which is necessary to favor the virion cellular access to dysregulate glandular function that could extend the effects beyond the acute phase of SARS-CoV-2 infection into the post-acute phase [29]. In addition, serum high-mobility group box 1 (HMGB1), a damage-associated molecular pattern and a mediator of severe inflammation, has been described to be elevated in severe COVID-19 patients and proposed as a marker of COVID-19 cytokine storm in immune cells [40].

In cytokine storm syndrome, the overactivation of T-lymphocytes entails a release of proinflammatory cytokines into the circulation, resulting in a positive feedback loop between the immune activation and the myocardial damage. A central role of interferon-gamma affecting the mitogen-activated protein kinase (MAPK) and Janus kinase (JAK)—a signal transducer and activator of transcription (STAT) signalling pathways with adverse cardiac events—has been described [41]. The endothelial disturbance created by viral invasion and the circulating cytokines could promote the appearance of microvascular disease [42]. Endothelial cell biomarkers, including von Willebrand factor (vWF) antigen, vWF pro-peptide, and factor VIII, are significantly elevated in convalescent COVID-19, suggesting a persistent endotheliopathy [43]. The cytokines can stimulate many processes involved in the activation of immune cells because of changes in the vascular environment promoting greater adhesion and blood with development of thromboembolic states on cardiovascular, cerebrovascular, and pulmonary tissues [33]. Pulmonary fibrosis can be developed via increasing concentrations of the fibrosis-promoting angiotensin 2 [44].

Pulmonary hypertension, pulmonary embolism, and pulmonary fibrosis are common in long-COVID-19 patients, resulting in impaired lung function. With the change in lung function, chronic hypoxia occurs, and hypoxia-induced inflammation may exacerbate capillary dysfunction and promote thrombosis as a vicious cycle. Chronic hypoxia leading immune cells could produce more inflammatory cytokines and exacerbate capillary dysfunction. It is also known that chronic persistent inflammation in long-COVID-19 patients may stimulate platelets, endothelial cells, and other inflammatory cells, promote the upregulation of procoagulant factors, and destroy the protective function of the vascular endothelium, causing abnormal coagulation. Therefore, the inflammation causes thrombosis, and the resulting blood clots can directly contribute to inflammation [1].

Heparanase is increased in COVID-19 patients and is related to its pathogenicity through factor X activation [10]. The potential impact of heparanase activity and endothelial damage in COVID-19 disease have been proposed, suggesting heparanase as a biomarker to predict clinical course [45]. In a series of 277 autopsies of patients who died due to COVID-19, the presence of microthrombi, macrothrombi, inflammation, and/or intraluminal megakaryocytes was up to 47% [46]. Viral inclusions in endothelial apoptotic cells and microvascular lymphocytic endotheliitis, with the infiltration of inflammatory cells around the vessels and endothelial cells, have also been reported [47].

## 4. Postural Orthostatic Tachycardia Syndrome and Pathological Mechanism: Hypercoagulability, Autoimmunity, and Endothelial Dysfunction

There is evidence from the scientific literature about the different types of cardiovascular autonomic dysfunction developing during and after COVID-19, with postural orthostatic tachycardia syndrome (POTS) being the most frequent diagnosis in individuals with post-COVID-19 orthostatic complaints [48]. For this reason, we believe they deserve a special section.

There are several reports regarding specific complications arising from POTS and thromboembolism or hypercoagulability [49,50,51,52]. In fact, the term orthostatic hypercoagulability has been proposed [51], associated with elevated vWF and factor VIII activity [51,52,53].

Plasma proteomic profiling in POTS reveals new disease pathways, with the strongest network interactions, particularly for proteins, involved in thrombogenicity and enhanced platelet activity (upregulation of glycoprotein 1B, downregulation of cartilage oligomeric matrix protein, upregulation beta parvin) but also inflammation, cardiac contractility, and hypertrophy and increased adrenergic activity [50]. Authors have indicated that thromboprophylaxis with low-molecular-weight heparins (LMWHs) merit further investigation, focusing on proteins associated with the von Willebrand complex, such as the involvement of glycoprotein 1B [50]. Thrombin ligation to glycoprotein 1b is inhibited by heparin [54]. On the other hand, other authors have suggested that elevated norepinephrine, epinephrine, plasma renin, and cortisol levels are associated with blood hypercoagulability during standing [55]. The hypercoagulability state in the orthostatic intolerance of the blood has been attributed to both hemoconcentration and endothelial-activated coagulation [49].

The presence of various autoantibodies has been reported in COVID-19 disease, and their association with the disease course and the durability of some related symptoms have been explored. Lupic anticoagulant activity, immunoglobulin G or M anti-cardiolipin/anti-phospholipid antibodies, anti-platelet factor 4 autoantibodies, anti-heparin, anti-ACE2, anti-annexin, anti-nuclear antibodies, anti-endothelial antibodies, antiganglioside, antimuscarinic, and antiadrenergic antibodies have been detected in COVID-19 patients. When these antibodies are presented, their biological effects persist for some time, inducing the switch to long-lasting autoantibodies. Currently, the evolution to a chronic autoimmune disease remains poorly understood [56].

POTS is associated with autoantibodies against alpha y beta-adrenoceptors and muscarinic receptors, known to be able to disturb the balance of neuronal and vascular processes [57,58,59,60,61,62]. Interestingly, cytosolic heparin inhibits muscarinic and α-adrenergic Ca^2+^, which are released in smooth muscle [63].

Other recognized autoantibodies in POTS include ganglionic neuronal nicotinic acetylcholine receptor (g-AChR), anti ECA2 circulating anti-nuclear, anti-thyroid, anti-N-methyl-D-aspartate-type glutamate receptor, anti-opioid like 1 receptor, anti-cardiac protein, anti-phospholipid, and Sjögren’s antibodies [62,64].

Recent studies suggest that POTS is characterized by underlying endothelial dysfunction [65] and low-grade inflammation in small vessels [66]. A correlation is presented between endothelial function and the autonomic nervous system and vasomotor activity, forming a complex triangle in the pathogenesis of dysautonomia [67].

## 5. Potential Beneficial Effects of Heparin in COVID-19 Post-Acute Sequelae: Immunomodulatory, Anticoagulant, Antiviral, Endothelial, and Vascular Resistance Effects

An ideal drug in COVID-19 progression should be to target the over-activation of the immune responses, including the block of the interaction between IL-6 and IL-6 receptor, the control of cytokine release storm from leukocytes, the inhibition of the formation and the clearance of NETs, the suppression of complement activation, and the control of inflammation after affection [12].

Heparin is structurally related to heparan sulfate, a negatively charged glycosaminoglycan that serves as binding sites for growth factors, cytokines, selectins, extracellular matrix molecules, and viruses, including SARS-CoV-2 [68,69]. Heparin binds to cytokines by competing with membrane-associated heparan sulfate and causes a decrease in cytokine concentration on the cell surface. Heparin can also occupy the receptor binding site on cytokines, partially preventing the interaction between interferon-gamma and its receptor [70]. Heparin also attenuates TNF-alpha-induced inflammatory response through a CD11b-dependent mechanism [71] and induces a stable complex between IL-6 and heparin [72]. Heparin also inhibits angiotensin-2-induced vasoconstriction on isolated mouse mesenteric resistance arteries [73] and protects against angiotensin-2-induced cardiac remodeling via the attenuation of oxidative stress in mice [74]. Other actions include the interferences between heparin with classical and alternative complement pathways by inhibiting the formation of several complement factors such as active C1 complex and C3 convertase [75] and reducing the endothelial cell damage induced by neutrophil extracellular traps [76].

Heparin possesses antifibrotic activity, which is mediated by the cellular secretion of HGF [77]. High plasma levels of fibroblast growth factor 23 have been associated with an increased risk of COVID-19 [78]. Heparin could potently modulate the receptor binding of growth factors such as fibroblast growth factor (FGF), vascular endothelial growth factor, and heparin-binding epidermal growth factor (HB-EGF). Heparin-binding growth factors modulate diverse biological activities including cellular proliferation, cellular differentiation, morphogenesis, and angiogenesis [79].

In a systematic review supported by several clinical trials, heparin was associated with relevant anti-inflammatory effects in patients with inflammation-induced conditions such as asthma or in subjects who had undergone surgical procedures such as cardiopulmonary bypass and cataract surgery [80], showing its potential anti-inflammatory effect.

With regards to its anticoagulation effect, heparin is quite well known as an anticoagulant through the inhibition of blood coagulation factors, particularly factors Xa and IIa (thrombin). Heparin activates antithrombin, an endogenous serine protease inhibitor regulating coagulation [81].

COVID-19 patients develop a profound depression of the fibrinolytic system against the background of hypercoagulation activation. Several studies have reported that the formation of micro clots in blood is the main mechanism of the so called long COVID-19 [13,14,18,22,28].

LMWHs are potent inhibitors of heparanase, thrombus, and inflammation, and they also neutralize histones [81]. Histone and heparanase levels could explain interindividual sensitivities to heparin in COVID-19 patients [82]. Heparanase inhibition in SARS-CoV-2 might be beneficial in healing the coagulation system and in attenuating viral attachment and spread [83].

Heparin resistance associated with elevated factor VIII has been described [84], and it is noteworthy that this factor was found to be significantly elevated in convalescent COVID-19 patients [43].

One of the theories of PASC is viral persistence in the tissues [29]. The reason why heparin has been proposed as an antiviral is its binding to the spike glycoprotein of SARS-CoV-2, which inhibits infection [85]. Heparin has also been implicated in the inhibition of SARS-CoV-2 replication in human nasal epithelial cells [86]. Other mechanisms associated with the antiviral properties of heparin include its competition with SARS-CoV-2 for the binding to heparan sulfate, inhibiting the virus attachment to the cell surface and the viral entry [87], and its binding to SARS-CoV-2 Mpro, the enzyme for viral replication and transcription [88]. Finally, the activation of antithrombin by heparin or fondaparinux could increase the anti-TMPRSS2 and anti-SARS-CoV-2 activity of antithrombin [89].

It is known that severe COVID-19 patients present endotheliitis [12] and long-COVID-19 patients may present persistent endothelial dysfunction [90]. Heparin can enter the bloodstream in the early stages of pathological processes, preventing damage to the vascular endothelium by binding to and the inactivation of biologically active substances, reducing the endothelial adhesiveness, restoring the altered membranes electronegative potential, and inhibiting the activation of cellular and plasma coagulation factors [91]. All these mechanisms support the protective effects of heparin against endothelial dysfunction.

The primary pharmacological target of heparin could possibly be the endothelial glycocalyx, which regulates vascular tone and barrier integrity, preventing leukocyte adhesion and thrombosis [92].

The activity of heparanase has been associated with disease severity in COVID-19 patients, and LMWHs could reduce its activity [93]. The inhibition of heparanase by a non-anticoagulant heparin fragment prevented glycocalyx destruction in response to COVID-19 serum treatment [94,95].

Heparin has also been described to inhibit HMGB-lipopolysaccharide. Its use can reduce the damage to the cardiac microcirculation and the prothrombotic state [96]. Finally, the interaction of heparin with basic fibroblast growth factor could allow for the inhibition of smooth muscle cell proliferation in injured arteries, reducing the risk of endothelial dysfunction [97].

Regarding vascular resistance, data have demonstrated the participation of M3 receptor and integrin in the heparin-dependent relaxation of vascular smooth muscle [98]. Additionally, heparin inhibited angiotensin-2-induced vasoconstriction on isolated mouse mesenteric resistance arteries through Rho-A- and PKA-dependent pathways [73]. In addition, enoxaparin restored the altered vascular reactivity of resistance arteries in aged and aged–diabetic hamsters, and this effect could be of relevance to improving perfusion in the microvasculature [99].

Another unknown aspect, yet potentially relevant to establishing the role of heparin in treating long-term complications associated with COVID-19, is the differential role associated with various mutations of SARS-CoV-2. Extensive registries have described the association between various mutations and the case fatality ratio and the number of deaths per million [100]. Based on these findings, it is reasonable to hypothesize that other studies evaluating the relationship between the various mutations described in SARS-CoV-2 and the development and progression of long-term complications could prove highly beneficial. Attaining this novel pathophysiological knowledge of long COVID-19 could also be applied to elucidate the role of heparin as a treatment for this condition.

The mechanisms connecting post-acute sequelae with the potential beneficial effects of heparin are summarized in Table 1.

Although multiple potential beneficial effects of unfractionated heparin and LMWH targeting COVID-19 have been proposed, their adverse effects should be taken into account. In this context, one of the most relevant adverse effects associated with the use of heparin is the presence of thrombocytopenia. It consists of a decrease in platelet levels >30% at 5–14 days after starting heparin. The presence of antibodies that recognize complexes formed by platelet factor 4 and polyanions and antiprotamine is implicated in its pathogenesis. Among the studies evaluating this clinical complication, we can highlight the meta-analysis by Uaprasert et al., which demonstrated a pooled incidence of heparin-induced thrombocytopenia of 0.8%, which was higher in critically ill COVID-19 patients (2.2%) and lower in non-critically ill patients (0.1%). Similarly, the incidence of thrombocytopenia was 1.2% for a therapeutic dose and 0.1% for a prophylactic dose. The authors of the study considered that the overall observed incidence was similar to that previously reported for patients with medical conditions other than COVID-19. Among the 17 cases of heparin-induced thrombocytopenia with available clinical follow-up and confirmation diagnosis through platelet activation assays, a short-term mortality rate of 4.17% was reported [101]. We should consider that SARS-CoV-2 may also induce thrombocytopenia and hyperactivated platelets.

To adequately measure the risks associated with heparin in COVID-19, it is highly useful to establish the attributable impact of treatment with this pharmacological group on the incidence of bleeding. Few studies provide quality information in this regard, especially post hospital discharge. A retrospective analysis of 1171 patients hospitalized for COVID-19 and treated with prophylactic anticoagulation observed, in a 35-day follow-up after discharge, an incidence of major or clinically relevant bleeding of 0.9%. Statistically significant differences were observed between patients who continued anticoagulation after discharge and those who discontinued it (3.0% vs. 0.6%, *p* = 0.019) [102].

The role of a prophylactic dose of heparin versus a therapeutic dose is still a subject of debate in the scientific community [103,104]. To summarize this complex controversy through the greatest possible evidence, we highlight the findings of two meta-analyses published in 2023. The first of these examined the survival of hospitalized patients with non-critical COVID-19 disease. After pooling data from 3297 participants across six randomized clinical trials, the treatment with full-dose heparin-based anticoagulation versus a prophylactic or intermediate dosing showed a risk ratio of 0.76 (95% CI 0.59–0.98; *p* = 0.037) [7]. The second meta-analysis included critically ill patients from both randomized studies and prospective registries without a control group. The dose of heparin showed no significant influence on short-term mortality, deep vein thrombosis, arterial thrombosis, or bleeding incidence [103].

Choosing the right treatment with an adequate safety and benefit profile seems to be essential. In a large observational study, patients who received chronic anticoagulants (vitamin-K antagonist or direct oral anticoagulants) before COVID-19 infection did not show any reduction in mortality [105]. The lack of effects on immunothrombosis of these anticoagulants could probably explain it. They have been shown to reduce macrovessel thrombosis but were not effective in ameliorating the microvessel thrombosis in COVID-19 [106].

## 6. Conclusions and Future Directions

COVID-19 PASC are mediated by several physiopathological mechanisms, including hypercoagulability, autoimmunity, and endothelial dysfunction. One frequent sequela presented in post-COVID-19 patients is postural orthostatic tachycardia syndrome. The mechanisms directly associated with PASC determine the thrombotic consequences of COVID-19 and the need for early anticoagulation therapy. Heparin has been described as a treatment with several potential beneficial properties in COVID-19 PASC beyond its anticoagulant properties, such as immunomodulatory, antiviral, endothelial, and vascular resistance effects though different mechanisms. These properties could be helpful in the treatment of COVID-19 post-acute sequelae. Choosing the right treatment with an adequate safety and benefit profile appears essential in improving adverse outcomes in patients with COVID-19. Further investigation into the potential benefits of antithrombotics in COVID-19 PASC is certainly necessary. However, to the extent of our knowledge, there are currently no ongoing randomized clinical trials aimed at evaluating the effect of anticoagulation on the prevention or progression of long COVID-19. Probably, until these randomized studies are conducted, we will not have sufficient-quality evidence to address the fundamental questions addressed in this review.

## Figures and Tables

**Table 1:** Mechanisms connecting post-acute sequelae with potential beneficial effects of heparin.

Mechanisms	SARS-CoV-2	Heparin
Antiviral effects	Association between PASC and the viral persistence in the tissues.	Binding to spike glycoprotein. Competition with SARS-CoV-2 for the binding to heparan sulfate.
TMPRSS2	Necessary to provide the virion cellular access.	Activation of antithrombin, and antithrombin binds inhibition.
Interleukin-6	Increased release.	Inhibition.
Interferon-gamma	Increased levels.	Prevention of the interferon-gamma—interferon-gamma receptor interaction.
TNF-alpha	Production.	Attenuation through a CD11b-dependent mechanism.
Complement	Activation.	Inhibition of activation.
Angiotensin 2	Negative regulation of ACE2 activity. Protection against angiotensin-2-induced cardiac remodeling. Vasoconstriction.	Inhibition of angiotensin-2-induced vasoconstriction.
HMGB-lipopolysaccharide	Elevation.	Inhibition.
Heparanase	Increase.	Inhibition.
Heparan sulfate	Interaction of spike glycoprotein’s receptor-binding domain with heparan sulfate.	Binding of heparan sulfate, inhibition of the virus attachment to the cell surface and the viral entry.
Histones	Increase.	Neutralization.
Pulmonary fibrosis	Increased concentration of the fibrosis-promoting angiotensin 2. Participation of TGF-beta pathways in the development process of pulmonary fibrosis.	Antifibrotic activity, mediated by cellular secretion of hepatocyte growth factor. Activation of TGF-beta by dissociating it from alpha 2-macroglobulin.
Endothelial function	Endothelial dysfunction.	Reduction in endothelial adhesiveness. Restoration of the altered membrane’s electronegative potential. Inhibition of the activation of cellular and plasma coagulation factors. Glycocalyx-stabilizing effect.
Neutrophil extracellular traps	Increased.	Reduction in endothelial cell damage.
Muscarinic and adrenergic receptors	Presence of muscarinic acetylcholine receptor antibody.	Inhibition of muscarinic and α-adrenergic calcium release in smooth muscle.
Hepatocyte growth factor	Upregulation.	Antifibrotic activity, mediated by cellular secretion of hepatocyte growth factor.
Glycoprotein 1B	Upregulation.	Inhibition of thrombin ligation to glycoprotein Ib.
Coagulation	Hypercoagulation, platelet hyperactivity, and abnormal fibrinolysis. Increase in vWF antigen, vWF pro-peptide, and factor VIII.	Anti-Xa activity. Factor IIa inhibition. Decrease in vWF synthesis.

ACE2: angiotensin-converting enzyme 2; HMGB: high-mobility group box; PASC: post-acute sequelae; SARS-CoV-2: severe acute respiratory syndrome coronavirus 2; TMPRSS2: transmembrane serine protease 2; TGF: transforming growth factor; TNF: tumor necrosis factor; vWF: von Willebrand factor.
